# Sustainable Aquaculture: Nutrition Studies in Early Developing Finfish, Ornamentals and Experimental Model Fish

**DOI:** 10.3390/ani12111384

**Published:** 2022-05-27

**Authors:** Miquel Planas, Ike Olivotto

**Affiliations:** 1 Department of Marine Ecology and Resources, Institute of Marine Research (CSIC), 36208 Vigo, Spain; 2 Department of Life and Environmental Sciences, Marche Polytechnic University, 60131 Ancona, Italy; i.olivotto@staff.univpm.it

Enhancing the knowledge of feeding/nutritional requirements is key in the growth of fish development and for the optimization of rearing techniques. Fish rearing has largely progressed in recent years for new species, but there is still a need for feeding optimization of already established species and the development of adequate feeding schedules in novel cultured species, especially in early developmental stages. Among other factors, feed optimization should rely on the enhancement of feeding efficiencies, the identification and implementation of alternative and sustainable feed ingredients or functional additives, and the preservation of fish welfare. Due to the growing demand for dietary sources, there is a need for novel information to improve production efficiencies but also to sustain environmental conditions.

The Special Issue “Sustainable Aquaculture: Nutrition Studies in Early Developing Finfish, Ornamentals and Experimental Model Fish” aimed to provide novel knowledge on commonly reared finfish species, ornamentals and species used as biological models, mostly on feeding performance, new feeding strategies, novel and sustainable dietary ingredients for aquafeed production, microdiets, stress response, and welfare. Due to the implications of both the environment and the feeding on the chemical composition of fishes, the need of keeping track of outputs (traceability) is essential to comply with trade regulations.

Most research studies published in the Special Issue were focused on the use of feed additives or alternative dietary ingredients for the enhancement of fish welfare and rearing performance.

Prokešová et al. [1] tested the effects of four levels (0, 1, 3, and 6% *w*/*w*) of humic substances (HS) as natural immunostimulants and growth promoters in juvenile African catfish (*Clarias gariepinus*) during a 56-day exposure. Although growth was not improved by HS addition, moderately positive effects were observed regarding health status and antioxidant parameters, especially in the group receiving 3% HS.

Montaser et al. [2] studied for eight weeks the effect of *Boswellia serrata* resin extract (BSRE) as a feed additive (0, 5, 10, or 15 g kg^−1^) on the growth performance, immune response, antioxidant status, and disease resistance of Nile Tilapia (*Oreochromis niloticus*) (initial weight: 21.82 ± 0.48 g). The fish were fed on one of four treatments with four levels of BSRE. After the end of the feeding trial, the fish were challenged with *Staphylococcus aureus*. The histoarchitecture of many vital organs were affected by the highest levels of BSRE, but the level of 5 g kg^−1^ BSRE improved fish growth without causing harmful effects on fish health. The authors concluded that BSRE addition can enhance the antioxidant activity, immune status, and disease resistance of Nile tilapia to *Staphylococcus aureus*.

Xavier et al. [3] assessed the improvement of digestive maturation and robustness of gilthead seabream (*Sparus aurata*) larvae submitted to dietary curcumin supplementation (0—control, 1.5 or 3.0 g kg^−1^ feed for 27 days). Curcumin supplementation seemed to promote digestive capacity and modulate the oxidative status during ontogeny.

Das Neves et al. [4] evaluated the inclusion of fumaric acid (FA; 0, 5, 10, 15, 20, and 30 g kg^−1^) in diets of Nile tilapia juveniles over 35 days. The inclusion of 15 g kg^−1^ of FA was effective in promoting growth, feed efficiency, and protein efficiency ratio, improving intestinal morphometry, and decreasing Gram-negative bacteria in the guts. The enhancement was proportional to fumaric acid supplementation of up to 14–15 g kg^−1^, followed by a reduction thereafter.

Bae et al. [5] performed an 8-week feeding trial to evaluate several combinations of dietary soluble extract hydrolysates from fishery by-products, such as shrimp soluble extract (SSE) with or without inosine monophosphate (IMP), tilapia soluble extract (TSE) and squid soluble extract (SQSE), in *Nile tilapia* juveniles. The results attained in this study suggested that dietary shrimp soluble extracts might improve the growth performance, non-specific immune responses, and disease resistance in juvenile *Nile tilapia*. Contrarily, inosine monophosphate did not enhance the effects of shrimp soluble extracts.

Zarantoniello et al. [6] demonstrated that nutritional programming through parental feeding may make it possible to extend the fish meal substitution level (0, 25, 50, 75 and 100%) with full-fat Black Soldier Fly (*Hermetia illucens*; BSF) prepupae meal in the diet of zebrafish (*Danio rerio*) up to almost 100% without incurring the well-known negative side effects of BSF-based diets. The effects of programming via broodstock nutrition on F1 zebrafish larvae development were investigated. The results (biometric, gas chromatographic, histological, and molecular analyses) evidenced that the same BSF-based diets provided to adults were able to affect F1 zebrafish larvae fatty acid composition without impairing growth performances, hepatic lipid accumulation and gut health. Groups challenged with higher BSF inclusion with respect to fish meal (50, 75 and 100%) showed significant downregulation of stress response markers and a positive modulation of inflammatory cytokines gene expression.

Kasprzak et al. [7] contributed to the welfare of house-kept ornamentals with a 12-week trial to assess the effects of two commercial flakes and a mix of lyophilized natural food on the condition of co-reared ornamental neon tetras (*Paracheirodon innesi*) and glowlight rasboras (*Trigonostigma hengeli*). Histological observations and measurements of digestive organs showed pronounced differences between the two species, with severe lipoid liver degeneration in glowlight rasboras fed on the natural food. The authors highlighted that the nutritional strategy applied for a community tank may yield radically divergent effects in co-housed and anatomically diverse ornamental fish species, potentially leading to effects that might remain unnoticed by simple external body observations and measurements.

Shafique et al. [8] reviewed an update on the potential of yellow mealworms (*Tenebrio molitor*) as an alternative ingredient in the partial or complete substitution of fishmeal in diets or cultured organisms. The use of alternative ingredients in the aquafeed industry would contribute to reducing the increasing demand and rising cost of fishmeal. Although yellow mealworms have some undesirable nutritional characteristics, the authors evaluated and discussed the effects of *T. molitor* on growth, biometric indices, and body composition in finfish and shellfish species. The authors also assessed the use of fish fed *T. molitor*-based diets on hematological parameters, immunological responses, antioxidative efficacy, intestinal health status, and sensory criteria. Future studies would be implicating to digestion and absorption processes using more descriptive and qualitative tools.

Paray et al. [9] reviewed the use of yucca (*Yucca schidigera*) extract as a practical solution for the regulation of ammonia accumulation in fishponds or intensive aquaculture systems. From the relevant published data, it is concluded that yucca could enhance growth, survival, blood biochemical quality, immunological indices, and the antioxidative capacity of aquatic animals. The review highlights the higher overall performances of aquatic organisms treated with yucca as a dietary additive or a water cleaner, contributing to a more sustainable aquaculture activity.

Fish farmers may apply short- or long-term fasting or limited feeding strategies to reduce mortality or stress under certain circumstances (e.g., water quality degradation, disease outbreaks, sorting, harvesting, or cost reduction). Kim et al. [10] determined the effects of varying durations of post-fasting satiation feeding on the feed utilization, body composition, blood composition and compensatory growth in juvenile Leopard Mandarin (*Siniperca scherzeri*) fish exposed to food deprivation. The juveniles were fasted for 5–14 days and achieved full compensatory growth after 4 weeks of re-feeding, showing morphological/biochemical indices and body and blood composition similar to the control group. The ultimate goal of the study was to develop an effective and economically optimal feeding supply strategy in *S. scherzeri* farms.

The potential of seahorses (*Hippocampus* spp.) as ornamental species has increased in recent years as a consequence of (or in parallel to) the development of new rearing techniques. The main bottleneck in the rearing of seahorses is located in the early developmental stages. Valladares and Planas [11] provided a study on the relationship between nutrient assimilation and the effects on initial mortalities and growth in *H. guttulatus* following a stable isotope (δ^13^C) approach. For that, the early feeding on copepods or *Artemia* nauplii was evaluated, and differences in the assimilation efficiency of preys offered were discussed. Both growth and survival in juveniles fed on copepods were enhanced compared to those fed on *Artemia*. This result is in agreement with the low digestion capability in seahorse juveniles. The study by Hernández-Urcera et al. [12] provided new insights into the understanding of growth and food assimilation in *H. reidi* juveniles following a laboratory diet switch. This study highlighted the relevance of feeding on copepods and their effect on isotopic patterns and discrimination factors in seahorse juveniles after a dietary shift. The isotopic analysis revealed that almost all tissue turnover was attributed to growth, especially with expended feeding on copepods. The authors recommended a long period of feeding on copepods during the first days of development. The study also provided for the first time diet–tissue discrimination factors in seahorse juveniles.

The global market of dried seahorses still relies on blurry trade chains that often cover less sustainable practices. As such, reliable tools that allow the enforcement of traceability, namely, to confirm the geographic origin of traded seahorses, are urgently needed. Cabral et al. [13] provided a preliminary evaluation of the use of elemental fingerprints in different bony structures of long-snouted seahorses *H. guttulatus* raised in captivity in two different locations (southern Portugal and Northern Spain) to discriminate their geographic origin. The contrasting elemental fingerprints of *H. guttulatus* raised in the two locations allowed their reliable discrimination. This contribution was a promising forensic approach to discriminate the geographic origin of seahorses raised in captivity, but it should be validated for wild conspecifics originating from different locations, as well as for other seahorse species.

Finally, the Editors acknowledge all researchers that successfully contributed their studies to this Special Issue.
